# Chronic recurrent wheals – If not chronic spontaneous urticaria, what else? 

**DOI:** 10.5414/ALX02375E

**Published:** 2023-01-03

**Authors:** Hanna Bonnekoh, Karoline Krause, Pavel Kolkhir

**Affiliations:** 1 Institute of Allergology, Charité – Universitätsmedizin Berlin, corporate member of Freie Universität Berlin, Humboldt-Universität zu Berlin, and Berlin Institute of Health, and; 2 Fraunhofer Institute for Translational Medicine and Pharmacology ITMP, Allergology and Immunology, Berlin, Germany

**Keywords:** chronic spontaneous urticaria, wheals, urticarial rash, urticarial vasculitis, Schnitzler syndrome, cryopyrin-associated periodic syndrome, autoinflammation, differential diagnosis

## Abstract

Chronic urticarial rash, mostly due to chronic spontaneous urticaria (CSU), is seen in up to 1 – 4% of the general population. Urticarial vasculitis (UV) and autoinflammatory syndromes, i.e., cryopyrin-associated periodic syndromes (CAPS) and Schnitzler syndrome (SchS), can mimic CSU-like rash but represent rare disorders with systemic symptoms including fever, headache, conjunctivitis, and arthralgia. Clinical and laboratory features can point to the presence of any of these diseases in patients initially presenting with chronic urticarial rash. These include long-lasting wheals (> 24 hours), lesional burning, systemic symptoms, and/or increase in inflammatory markers (e.g., C-reactive protein, serum amyloid A, and/or S100A8/9). Lesional skin biopsy usually demonstrates leukocytoclastic vasculitis (UV) or neutrophil-rich infiltrate (CAPS and SchS). In contrast to CSU, where second-generation H1 antihistamines and omalizumab allow to control symptoms in most patients, systemic immunosuppression and anti-interleukin (IL)-1 therapies are needed in case of UV and autoinflammatory diseases, respectively. The rarity and low awareness of CSU differential diagnoses may be related to the longer delays in diagnosis and therapy in those affected with UV, CAPS, and SchS. Knowledge of the differential diagnoses of CSU is important because only correct diagnosis allows adequate therapy. Complications such as the development of lymphoproliferative disease in SchS and amyloidosis in CAPS, and the presence of comorbid diseases, such as systemic lupus erythematosus in UV, must be considered and monitored.

## Recurrent wheals – What’s behind it? 

**Figure 1.**
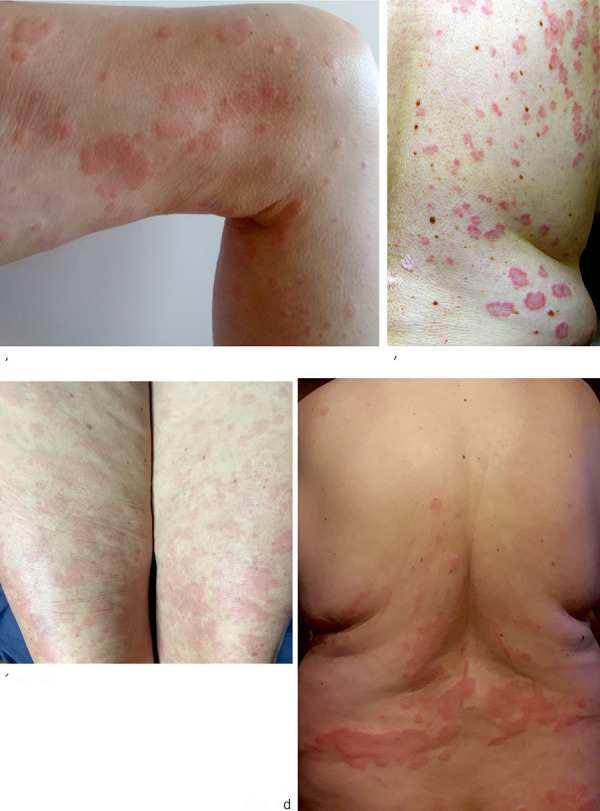
a: Wheals on the thigh in a patient with chronic spontaneous urticaria; b: Wheals with hyperpigmentation in urticarial vasculitis; c: Urticarial exanthema on the thighs of a patient with cryopyrin-associated periodic syndrome; d: urticarial exanthema on the trunk of a patient with Schnitzler syndrome.

Urticaria is a frequent clinical symptom in daily practice of dermatologists and allergists. Chronic recurrent urticaria, i.e., urticaria that persists for more than 6 weeks, is in most cases due to chronic spontaneous urticaria. Chronic spontaneous urticaria is a common skin disease with a worldwide prevalence of ~ 1 – 4% and leads to a significant reduction in quality of life [1]. Patients present with multiple itchy wheals (Figure 1a) and/or angioedema, often occurring daily without an identifiable trigger. Pathophysiologically, chronic spontaneous urticaria is a mast cell-mediated disease, and underlying autoimmune mechanisms with mast cell-activating antibodies (autoimmunity type I with IgE autoantibodies against endogenous antigens and autoimmunity type IIb with IgG anti-IgE/FcεRI antibodies) have been identified. According to the current guideline, standard treatment comprises second-generation H1 antihistamines at up to 4-fold doses. In case of insufficient symptom control with antihistamines, anti-IgE antibody omalizumab is approved for chronic spontaneous urticaria, which has proven to be safe and effective [2]. In addition to chronic spontaneous urticaria, there are also inducible forms of urticaria such as cold urticaria or symptomatic dermographism. Here, the wheals do not occur spontaneously but are triggered by specific physical stimuli such as cold or friction. Chronic inducible urticaria may also be associated with chronic spontaneous urticaria as a comorbidity. 

Importantly, urticarial vasculitis and autoinflammatory diseases can also present with wheals but have a distinct underlying pathogenesis and should be differentiated from ordinary chronic urticaria. Due to the rarity and the resulting lack of knowledge of the diseases, the diagnosis is often delayed and can lead to severe consequences of the diseases. 

## What indicates that chronic spontaneous urticaria may not be present and what is the best way to proceed? 

There are numerous clinical, laboratory, and therapeutic characteristics that may indicate that the chronic recurrent wheals conceal another diagnosis, in particular urticarial vasculitis or autoinflammatory disease. In principle, further diagnosis and re-evaluation of the diagnosis of chronic spontaneous urticaria should be performed in case of a clinically non-classical picture of urticaria. Systemic symptoms such as fever, joint complaints and fatigue, abnormal inflammatory markers, and an insufficient therapeutic response to antihistamines or omalizumab may indicate a different diagnosis (Table 1). 

### Medical history 

Taking a detailed history is very valuable and can lead to the correct diagnosis. The focus should be on the clinical symptoms, but also the response to treatment can be indicative, and often patients with chronic recurrent wheals have already received previous therapies. 10 important questions for patients with chronic recurrent wheals are listed in Table 2. 

### Symptom diary 

A symptom diary kept by the patient can facilitate the classification of clinical complaints and can support finding the correct diagnosis. 

### Clinical examination 

Inspection of the skin and a general physical examination are obligatory. Since it is not uncommon for the wheals not to be present during the medical presentation, it is advisable to review the patient’s photographic documentation and, if not available, to encourage them to take pictures of the skin lesions. 

### Diagnostics 

If patients report cold-associated wheals, it is recommended to perform a cold stimulation using the ice cube test or TempTest device. In contrast to classic acquired cold urticaria, this test is usually negative in patients with autoinflammatory diseases. 

### Laboratory tests 

According to the current international guideline, a differential blood count and C-reactive protein (CRP) and/or erythrocyte sedimentation rate (ESR) are recommended as basic screening tests in patients with chronic spontaneous urticaria [2]. If blood levels of inflammatory markers are elevated and/or leukocytosis or neutrophilia in the peripheral blood is present, testing of more sensitive inflammatory parameters is recommended. These include serum amyloid A (SAA), which is used as a disease activity marker and screening parameter for the presence of amyloidosis. In addition, S100A8/9, also called calprotectin, represents a general inflammation surrogate marker and serves to check the course of treatment. Urinalysis should be performed to exclude proteinuria, which may be indicative of AA amyloidosis. Serum electrophoresis with immunofixation and quantitative determination of immunoglobulins and light chains in the blood should be performed if Schnitzler syndrome is suspected. If there is clinical evidence of urticarial vasculitis, determination of complement factors C3, C4, C1q, and anti-C1q antibodies is also recommended to exclude hypocomplementemic urticarial vasculitis or hypocomplementemic urticarial vasculitis syndrome. 

### Genetics 

Genetic analysis should be performed if the patient’s history, clinical symptoms and laboratory parameters are highly suspicious for a hereditary autoinflammatory disease such as cryopyrin-associated periodic syndrome (CAPS). In a proportion of patients with autoinflammatory diseases, mutation detection is not successful despite the presence of a corresponding typical clinical picture. In CAPS, this may concern up to 40% of affected individuals. 

### Histology 

Lesional skin biopsy is recommended if wheals that persist for more than 24 hours and/or other accompanying symptoms such as fever or joint complaints are present. This should preferably be taken from the trunk of the body. Histological findings vary depending on the underlying disease (Table 1). 

## Overview of important differential diagnoses 

### Urticarial vasculitis 

Urticarial vasculitis is defined by the occurrence of long-lasting wheals (> 24 hours, often lasting several days) and the histopathologic finding of leukocytoclastic vasculitis [2]. The wheals are often accompanied by burning sensation rather than itching, may be painful, and often heal with hyperpigmentation (Figure 1b). In addition to wheals, angioedema may also occur. The clinical spectrum of urticarial vasculitis is broad and may be limited to skin symptoms; however, systemic symptoms such as fever, joint complaints, lymph node swelling, gastrointestinal, renal, and pulmonary involvement, ocular involvement, and neurologic symptoms may also occur. Patients with urticarial vasculitis are severely impaired in their quality of life [3]. Between 2 and 27% of patients who initially presented with urticaria were found to have urticarial vasculitis [4, 5, 6, 7, 8]. 

Urticarial vasculitis is a rare disease and the exact prevalence is unknown. Middle-aged women are more commonly affected by the disease. There may be possible underlying or associated diseases such as infections, malignancies, or autoimmune diseases such as systemic lupus erythematosus [9]. It is assumed that urticarial vasculitis is a type III hypersensitivity reaction with deposition of antigen-antibody complexes in the vascular lumina with subsequent activation of the complement system [10]. Based on complement consumption, normocomplementemic urticarial vasculitis (~ 80% of patients) differs from hypocomplementemic urticarial vasculitis (~ 9 – 21% of patients), which is often associated with increased clinical symptoms, or the even rarer hypocomplementemic urticarial vasculitis syndrome [4]. The histologic picture of urticarial vasculitis is characterized by erythrocyte extravasations and fibrin deposits in addition to leukocytoclastic vasculitis and can thus be distinguished from the histologic picture of chronic spontaneous urticaria [11]. The management of patients with urticarial vasculitis is a challenge worldwide due in part to the lack of guidelines and treatment algorithms [12]. To date, there are no approved treatments for urticarial vasculitis. In general, patients with urticarial vasculitis rarely respond to H1 antihistamines. Systemic steroids are shown to be effective but are not a long-term treatment option due to the severe side effect profile. Immunomodulatory and immunosuppressive drugs such as dapsone, hydroxychloroquine, colchicine, or azathioprine are frequently used, but all have limited effects. The use of anti-IgE omalizumab and interleukin-1 blockers anakinra and canakinumab in urticarial vasculitis showed a good response in single case reports and series, respectively, suggesting a complex pathophysiology of the disease [9]. 

### Autoinflammatory diseases 

Autoinflammatory diseases are characterized by multisystemic inflammation with no evidence of high-titer autoantibodies or antigen-specific T cells. Inflammation is primarily mediated by cells of the innate immune system, with the proinflammatory cytokine IL-1β playing a key role. In patients with autoinflammatory diseases, episodes of inflammation frequently affect the skin, the musculoskeletal system and internal organs [13]. Skin involvement may manifest clinically with chronic recurrent urticarial exanthema. Autoinflammatory diseases occur very rarely. Lack of awareness of the diseases and/or lack of knowledge of the clinical signs and symptoms among physicians often lead to a long delay in diagnosis of the affected persons. This can lead to irreversible long-term consequences, significantly reduced quality of life and inadequate treatment. 

**Cryopyrin-associated periodic syndrome**

The prototype of the hereditary autoinflammatory diseases is CAPS. It is a very rare disease with an incidence of ~ 1 – 3 patients per million people. Although the symptoms of CAPS appear in early childhood, the disease should always be considered for differential diagnosis in adults due to the lack of knowledge of the diagnosis and the possibility of mild courses of the disease. 

CAPS includes the three subtypes with increasing clinical severity: familial cold urticaria (FCAS), Muckle-Wells syndrome (MWS), and chronic infantile neurologic cutaneous articular syndrome (CINCA/NOMID) [14, 15]. Clinically, chronic recurrent wheals appear at the skin (Figure 1c). Patients often report cold as a trigger factor for skin symptoms. Depending on the severity of the disease, the episodes of inflammation may be short-lived (< 1 day) but may also persist chronically. The skin symptoms are accompanied by general symptoms such as weakness, fatigue, headache, fever, gastrointestinal and musculoskeletal complaints, and eye involvement, some of which occur daily. The main complication of persistent chronic inflammation is the development of AA amyloidosis with consecutive renal failure as well as neurological deficits such as sensorineural deafness and visual loss. The pathophysiological basis of CAPS is an autosomal dominant inherited gene defect with variable penetrance of the nucleotide-binding domain-like receptor protein 3 (NLRP3). This results in spontaneous secretion of interleukin (IL)-1β and consecutive inflammatory symptoms. In clinical studies, interleukin-1 inhibitors in CAPS have been shown to be very effective in reducing clinical symptoms and signs of inflammation and are therefore considered as standard treatment. Concomitant diseases such as hearing loss or amyloidosis can also be positively influenced by the treatment [16, 17]. 

**Schnitzler syndrome**

Schnitzler syndrome is also, with ~ 350 cases reported in the literature, a very rare disease and is considered to be an acquired interleukin-1-mediated autoinflammatory syndrome. The time to diagnosis of Schnitzler syndrome is long, taking a median of up to 5 years [18]. 

Schnitzler syndrome is characterized by the presence of chronic urticarial exanthema (Figure 1d) and monoclonal gammopathy (major criteria). According to the Strasbourg criteria, in addition to the two major criteria, two of the minor criteria must be present in case of IgM-type or IgG-type monoclonal gammopathy to confirm the diagnosis. The minor criteria include: recurrent fever (> 38 °C) without known cause, a neutrophil-rich infiltrate in the lesional skin biopsy, changes in bone structure, and elevated values of inflammatory markers (leukocytosis/elevated CRP) [19]. In addition, affected individuals report joint complaints, muscle and bone pain, headache, and fatigue. Lymphadenopathy and splenomegaly may occur. Men are affected slightly more often than women, and disease onset is around age 50. Systemic and cutaneous inflammation is also mediated, as in CAPS, by IL-1β produced in the skin by neutrophil granulocytes and mast cells [20, 21]. Targeted IL-1 blockade is shown to be effective and safe in the long term treatment of patients with Schnitzler syndrome, but it is not approved [22]. Approximately 20% of patients may develop a myeloproliferative disorder such as Waldenström’s disease or multiple myeloma during the course of the disease. As with CAPS, there is a risk of developing amyloidosis with persistent inflammation. 

A comparative overview of the characteristics to differentiate chronic spontaneous urticaria from urticarial vasculitis and autoinflammatory diseases CAPS and Schnitzler syndrome is shown in Table 1. 

## Other autoinflammatory diseases presenting with wheals 

There are other monogenetic but also polygenetic/acquired autoinflammatory diseases that may present with urticarial exanthema (Table 3) [23, 24]. The present review is limited to the detailed presentation of the two prototypes of urticarial autoinflammatory diseases. 

## Conclusion 

If chronic recurrent wheals occur, chronic spontaneous urticaria is the most probable diagnosis. However, differential diagnoses such as urticarial vasculitis and autoinflammatory skin diseases should be considered and further clarified in the presence of certain characteristics such as prolonged wheals, concomitant systemic symptoms, elevated blood levels of inflammatory markers and inadequate response to antihistamines. 

## Funding 

This research received no specific grant from any funding agency. 

## Conflict of interest 

H. Bonnekoh has received honoraria for lectures and advisory boards from Abbvie, Intercept Pharma, Novartis, Sanofi-Aventis, and Valenza Bio Inc. There is no conflict of interest regarding this article. 

K. Krause declares that there is no conflict of interest with regard to this article. 

P. Kolkhir has received honoraria for lectures and advisory boards from Roche, Novartis, and Valenza Bio Inc. There is no conflict of interest with respect to this article. 

**Table 1.** Characteristics to distinguish chronic spontaneous urticaria from important differential diagnoses.

Characteristics	Chronic spontaneous urticaria	Urticarial vasculitis	Autoinflammatory diseases	
			Cryopyrin-associated periodic syndrome	Schnitzler syndrome
Skin	Wheals and/or swellings	Wheals with/without swellings	Cold-associated appearance of wheals, partly maculopapular exanthema	Wheals
Duration of individual efflorescences	Transient, minutes to hours	> 24 hours in most cases	Hours to 24 hours	Hours to 24 hours
Localization of efflorescences	Rather asymmetric distribution, whole body	Whole body	Rather symmetrical, face less often affected	Rather symmetrical, often on the trunk of the body
Accompanying symptoms on the skin	Strong itching	Rarely itching, burning, pain postinflammatory hyperpigmentation	Burning	Burning
Occurrence of angioedema	Possible (~ 40 - 50%)	Occurrence possible	Not described	Very rare
Systemic complaints	Rare	Spectrum: joint pain, fever, muscle pain, gastrointestinal, pulmonary, renal involvement	Spectrum: depending on the degree of expression, including fever, fatigue, arthralgia/arthritis, ocular inflammation, headache	Fever, arthralgias, muscle/bone pain, fatigue
Age at onset of disease	Any age, in most cases > 30 years of age.	Midlife (~ 45^th^ year of life)	Since birth/early childhood but also possible in later adulthood in milder forms	50^th^ – 60^th^ year of life
Duration of disease	Mean/median: ~ 1 – 4 years	Mean/median: ~ 1 – 4 years	Lifetime	Usually since the onset of the disease, then lifelong
Gene/ mode of inheritance	–	–	NLRP3 autosomal dominant or sporadic	Complex
Possible consequences and complications	Severe impairment of quality of life	Severe impairment of quality of life, involvement of internal organs for example lungs, kidney	Severe impairment of quality of life, amyloidosis, sensorineural deafness, central nervous system involvement	Severe impairment of quality of life, lymphoproliferative disease, amyloidosis
Inflammatory markers	CRP may be slightly elevated, usually unremarkable	CRP, ESR elevated; In hypocomplementemic form: C3, C4 decreased, C1q antibodies	CRP, ESR, SAA, S100A8/9 elevated; leukocytosis with neutrophilia	CRP, ESR, SAA, S100A8/9 increased, leukocytosis with neutrophilia
Histology of the lesional skin	Dermal edema and sparse mixed-cell perivascular infiltrate	Leukocytoclastic vasculitis	Perivascular and interstitial neutrophil-rich infiltrate	Perivascular and interstitial neutrophil-rich infiltrate
Efficacy of antihistamines	Yes	Rare	No	No
Efficacy of omalizumab	Yes; approval	Case reports and series described	No	No
Efficacy of Interleukin-1 blockade	No	Case series described	Yes; approval for anakinra and canakinumab	No approval; interleukin-1 blockade effective
Characteristic features	Therapeutic response to H1 antihistamines or omalizumab	Healing of wheals with postinflammatory hyperpigmentation, histology with leukocytoclastic vasculitis.	Cold-related complaints	Monoclonal gammopathy

CRP = C-reactive protein; ESR = erythrocyte sedimentation rate; IL = interleukin; SAA = serum amyloid A.

**Table 2.** Ten important questions for patients with chronic recurrent wheals.

Question	Answer indicative for
1. Since when do the wheals appear?	If wheals appear since birth/early childhood, think of hereditary autoinflammatory diseases
2. How long do individual wheals persist? Here, the duration of the wheals should be inquired, especially with regard to the persistence of the skin changes beyond 24 hours.	Wheals lasting > 24 hours indicative of urticarial vasculitis
3. Does hematoma/dark spots appear when the wheals heal?	Occurrence of postinflammatory hyperpigmentation indicative of urticarial vasculitis
4. Are the wheals accompanied by itching and/or burning and/or pain?	Pruritus is a leading symptom of chronic spontaneous urticaria; burning sensation often occurs with wheals in autoinflammatory diseases; burning sensation and/or pain indicative of urticarial vasculitis
5. What is the diurnal course of the wheals?	Wheals in autoinflammatory diseases occur together with systemic complaints such as fever more frequently in the evening hours
6. Do the wheals occur episodically?	Relapsing appearance of wheals together with systemic symptoms common in autoinflammatory diseases
7. Are there physical factors, such as cold, that can trigger the wheals?	In cryopyrin-associated periodic syndrome, cold-associated wheals occur, but direct contact with cold does not usually trigger wheals, unlike classic cold urticaria
8. Besides the wheals, are there accompanying symptoms, such as joint pain, fever, fatigue?	In urticarial vasculitis, there are often accompanying symptoms; likewise in autoinflammatory diseases
9. Are there any relatives in your family (parents, siblings, children) who also report such complaints?	Indicative of hereditary autoinflammatory disorders such as cryopyrin-associated periodic syndrome
10. Was there any improvement in skin symptoms by taking H1 antihistamines?	Approximately 2/3 of patients with chronic spontaneous urticaria respond to therapy with H1 antihistamines; antihistamines are usually ineffective in urticarial vasculitis and autoinflammatory disease

**Table 3.** Further selected autoinflammatory diseases that may be associated with wheals.

Monogenetic autoinflammatory diseases	Complex autoinflammatory diseases
**With wheals ** – Familial Mediterranean fever (FMF)* – Hyper-IgD syndrome/mevalonate kinase deficiency (HIDS/MKD)* – Tumor necrosis factor (TNF) receptor-associated periodic syndrome (TRAPS)* – VEXAS (vacuoles, E1 enzyme, X-linked, autoinflammatory, somatic) syndrome* **With cold-induced wheals ** – NLRP12-associated cold-induced autoinflammatory syndrome (FCAS 2) – NLRC4-associated cold-induced autoinflammatory syndrome (FCAS 3) – PLCG2-associated antibody deficiency and immune dysregulation (PLAID) – Factor XII-associated cold-induced autoinflammatory syndrome (FACAS)	– Systemic juvenile idiopathic arthritis (SJIA)/adult onset Still’s disease (AOSD)* – Sweet syndrome*

*The appearance of wheals is not obligatory for these diseases.
